# Loss of Androgen Receptor-Dependent Growth Suppression by Prostate Cancer Cells Can Occur Independently from Acquiring Oncogenic Addiction to Androgen Receptor Signaling

**DOI:** 10.1371/journal.pone.0011475

**Published:** 2010-07-08

**Authors:** Jason M. D'Antonio, Donald J. Vander Griend, Lizamma Antony, George Ndikuyeze, Susan L. Dalrymple, Shahriar Koochekpour, John T. Isaacs

**Affiliations:** 1 Department of Oncology, The Sidney Kimmel Comprehensive Cancer Center, The Johns Hopkins University School of Medicine, Baltimore, Maryland, United States of America; 2 Department of Urology, The Brady Urological Institute, The Johns Hopkins University School of Medicine, Baltimore, Maryland, United States of America; 3 Departments of Biochemistry and Molecular Biology, Urology, Stanley S. Scott Cancer Center, Louisiana State University-Health Sciences Center, New Orleans, Louisiana, United States of America; 4 Department of Surgery, The University of Chicago, Chicago, Illinois, United States of America; University of Medicine and Dentistry of New Jersey, United States of America

## Abstract

The conversion of androgen receptor (AR) signaling as a mechanism of growth suppression of normal prostate epithelial cells to that of growth stimulation in prostate cancer cells is often associated with AR mutation, amplification and over-expression. Thus, down-regulation of AR signaling is commonly therapeutic for prostate cancer. The E006AA cell line was established from a hormone naïve, localized prostate cancer. E006AA cells are genetically aneuploid and grow equally well when xenografted into either intact or castrated male NOG but not nude mice. These cells exhibit: 1) X chromosome duplication and *AR* gene amplification, although paradoxically not coupled with increased AR expression, and 2) somatic, dominant-negative Serine-599-Glycine loss-of-function mutation within the dimerization surface of the DNA binding domain of the *AR* gene. No effect on the growth of E006AA cells is observed using targeted knockdown of endogenous mutant AR, ectopic expression of wild-type AR, or treatment with androgens or anti-androgens. E006AA cells represent a prototype for a newly identified subtype of prostate cancer cells that exhibit a dominant-negative AR loss-of-function in a hormonally naïve patient. Such loss-of-function eliminates AR-mediated growth suppression normally induced by normal physiological levels of androgens, thus producing a selective growth advantage for these malignant cells in hormonally naïve patients. These data highlight that loss of AR-mediated growth suppression is an independent process, and that, without additional changes, is insufficient for acquiring oncogene addiction to AR signaling. Thus, patients with prostate cancer cells harboring such AR loss-of-function mutations will not benefit from aggressive hormone or anti-AR therapies even though they express AR protein.

## Introduction

Within the last decade there has been a renewed interest in androgen receptor (AR) signaling, as it pertains to normal prostatic function, prostate carcinogenesis, and metastatic progression. In the normal prostate, AR functions via a reciprocal paracrine interaction between the epithelial and stromal cells [1]. Androgen binding to the AR in prostate stromal cells activates a transcriptional cascade resulting in the production and secretion of paracrine growth factors, known as andromedins, which diffuse into the epithelial compartment, bind cell surface cognate receptors, and activate signaling pathways that stimulate the proliferation and survival of the epithelial cells [1]. In the presence of physiological levels of androgen, and thus andromedins, ligand-bound AR located in the secretory luminal epithelial cell prevents the overgrowth of the epithelial compartment by suppressing cell proliferation and promoting cellular differentiation [1], [2], [3], [4]. The importance of this cell context-dependent AR growth-suppressive ability is documented by studies showing that conditional loss of AR expression in the epithelial compartment, but not in stromal cells, results in increased luminal epithelial cell proliferation [5], [6]. When a physiological level of androgen is not maintained, such as following androgen ablation, the level of andromedins decreases to a level where they can neither stimulate proliferation nor block the activation of apoptosis in the epithelial cells, and thus the prostate regresses [1].

During prostate carcinogenesis, both AR-independent and AR-dependent signaling mechanisms contribute to the malignant transformation of epithelial cells [7]. In the AR-independent pathway, AR protein is not expressed and therefore the AR-regulated suppression of malignant cell growth is lost. Importantly, when AR is then ectopically expressed in such AR-independent prostate cancer cells, androgen-activated AR signaling inhibits cell growth [8]. In the AR-dependent pathway, AR function is often converted from a growth suppressor to an oncogene stimulating prostate cancer cell survival and proliferation [1], [9], [10]. While either AR-independent or -dependent pathways are possible, the majority of prostate cancers acquire oncogenic AR signaling, thus providing the rationale for why androgen ablation is standard therapy for metastatic prostate cancer since it inhibits proliferation and activates apoptosis in these metastatic cancer cells [11]. Moreover, AR signaling remains a central target even for castrate-resistant metastatic prostate cancers [7]. This is based on the result of studies showing that, while uncommon in hormonally naïve patients, AR gene mutation and amplification, resulting in elevated AR protein expression, are detected in the majority of metastatic prostate cancer tissues obtained from patients with castrate-resistant metastatic disease [12], [13]. Consistent with these clinical observations, AR gene mutation, amplification and protein over-expression are commonly observed in the majority of prostate cancer cell lines derived from castrate-resistant hosts [14], [15]. These castrate-resistant prostate cancer cell lines do not undergo apoptosis when androgens are depleted or androgens antagonists are used; however, they stop proliferating and activate cell death if the AR protein level is reduced below a critical level both *in vitro* [14], [16], [17] and *in vivo* [18]. These observations are consistent with the concept of “oncogene addiction” [19] to AR protein expression and function, and document that such castrate-resistant prostate cancer cells remain addicted to AR signaling for their malignant growth [1], [9].

The presence of AR expression, somatic AR mutations or amplification does not necessarily mean, however, that a prostate cancer cell is addicted to AR signaling. This statement is based on the present study, which documents an additional subtype of human prostate cancer cells. In this subtype, AR has undergone a dominant-negative, loss-of-function mutation even though AR is genetically amplified without the patient ever receiving androgen ablation therapy. Such a dominant-negative loss of AR function in malignant cells produces a selective growth advantage by eliminating the normal androgen-dependent AR signaling-induced growth suppression; however, while necessary, it is insufficient for these malignant cells acquiring an oncogenic addiction to AR-signaling.

## Methods

### Ethics Statement

All animal studies were performed according to animal protocol MO09M434 approved by the Johns Hopkins Animal Care and Use Committee specifically for this study.

### Cells and Materials

E006AA [20], S006AA [20], LNCaP and PC-3 (obtained from ATCC, Manassas, VA)**,** and LNCaP C4-2B (obtained from UroCor, Oklahoma City, OK) cells were maintained in RPMI 1640 (Invitrogen, Carlsbad, CA), and LAPC-4 cells in IMDM (Invitrogen) plus 1 nM R1881, both containing 10% FBS (Hyclone, Logan, UT), 1× Pen/Strep, and L-glutamine. CWR22 xenograft tumor tissue was a kind gift from Thomas Pretlow (Case Western Reserve University, Cleveland, OH). Charcoal/dextran stripped FBS (csFBS) was obtained from Hyclone and used to supplement phenol red-free RPMI (Invitrogen) for luciferase and chromatin immunoprecipitation assays. The synthetic androgen R1881 was purchased from Perkin-Elmer (Boston, MA). PSA analysis was conducted by the JHMI Clinical Chemistry Laboratory. Cell growth was measured by a 3-(4,5-dimethylthiazol-2-yl)-2,5-diphenyltetrazolium bromide (MTT) assay (CellTiter Non-Radioactive Cell Proliferation Assay from Promega Corp. (Madison, WI)). Briefly, cells were plated in a 96-well format and allowed to adhere overnight. Cells were treated with vehicle or drug. At specific days, 15 µl of the MTT dye reagent was added to each well, plates incubated at 37°C for 4 hrs, 100 µl of Stop reagent was added to each well, and plates read by a Molecular Devices SpectraMAX plus plate reader at 570 and 650 nm. Absorbance readings were then plotted against a standard curve to determine cell numbers. Results are presented from day 4. Clonogenic survival was assayed as follows: 500 cells (parental E006AA, E006AA LV-non-silencing-shRNA, and E006AA LV-AR-shRNA) were plated per well in 6-well plates, allowed to adhere and colonize for six days, then simultaneously fixed and stained in a 0.5% crystal violet/25% methanol solution and colonies were counted manually.

### Karyotype Analyses and Fluorescence in Situ Hybridization

Cytogenetic analysis was performed using standard methods. Chromosomal abnormalities were described according to ISCN guidelines [21]. FISH was performed on the cell lines and on a normal male control using probes purchased from Vysis (Abbott Molecular), specific for the AR Gene (Xq12) and X centromere. The hybridization was done according to manufacturer's instructions. For each cell line, 100 interphase nuclei were scored and the ratio of red (AR) to green (Xcen) signals was calculated. A total of 9–20 metaphases were also analyzed. Images were acquired using a standard epifluorescence microscope and FISH View software (Applied Spectral Imaging).

### *In vivo* Tumor Assays

*In vivo* growth assays were performed as previously described [22], [23]. For subcutaneous injections, one million E006AA cells in 200 µl of 80% Matrigel (BD Biosciences, diluted with cold HBSS) were injected into the flanks of male Nude or NOG-SCID mice. For CWR22 xenograft inoculations, 20 mg of tissue was injected per mouse. Surgical castration was done as previously described [22], [23]. Passage of tumor tissue was conducted by mincing the tumor tissue, passing it through a sterile tissue strainer, washing it in PBS, and re-injecting 50 mg of tumor in 200 µl of 80% Matrigel.

### Immunohistological Detection

Immunostaining for AR (N-20; Santa Cruz Biotechnology, Santa Cruz, CA), PSA (Dako Cytomation, Carpinteria, CA), ΔNp63 (LabVison/NeoMarkers, Freemont, CA), Nkx3.1 (UMBC), and CK5 (Babco, Richmond CA) was done on xenograft sections as previously described [24], with the following modifications: Tissue sections were deparrafinized, rehydrated and briefly equilibrated in water. For AR and Nkx3.1 immunohistochemistry, antigen unmasking was performed by boiling slides in 1 mmol/L EDTA (pH 8.0) for 15 min. For p63 staining, slides were steamed for 20 min in Antigen Unmasking Solution (Vector Laboratories, Burlingame CA) for 20 minutes. Endogenous peroxidase activity was quenched by incubation with peroxidase block for 5 min at room temperature. For AR and Nkx3.1 staining, nonspecific binding was blocked by incubating in 1% bovine serum albumin in Tris-HCl pH 7.5 for 20 min at room temperature. Slides were incubated with primary antibodies including a rabbit polyclonal AR antibody (1∶25 dilution) for 45 min at room temperature; mouse monoclonal PSA antibody (1∶50 dilution) 45 min at room temperature; mouse monoclonal p63 antibody (1∶50 dilution) for 45 min at room temperature; rabbit polyclonal Nkx3.1 antibody (1∶1000 dilution) for 45 min at room temperature; and rabbit polyclonal CK5 antibody (1∶2500 dilution) for 45 min at room temperature. After primary antibody application, slides were incubated with poly-HRP conjugated secondary (EnVision (AR, p63) or PowerVision (PSA, Nkx3.1, CK5)) at 1∶3000 dilution for 30 min at room temperature. Signal detection was performed using 3,3′-diaminobenzidine tetrahydrochloride (DAB) as the chromagen for 20 min. Slides were counterstained with hematoxylin, dehydrated, and mounted.

### Lentiviral Infection

Stable wild-type human AR cDNA expression and FACS sorting for GFP-positive cells was done as previously described [8], [25]. Stable mutant LV-AR S599G was created first by PCR amplification of E006AA cDNA containing the S599G mutation. Both the PCR fragment and the wild-type lenti-viral AR plasmid were restriction digested with Eco81I and Tth111I. Digested PCR product and LV-AR plasmid were isolated, purified, ligated using rapid T4 ligase kit (Fermentas), and sequenced to confirm S599G insertion. Short-hairpin knockdown of AR was conducted using MISSION^TM^ lentiviral shRNA transduction particles targeting either the AR alone or a non-silencing shRNA control (Sigma-Aldrich, St. Louis, MO). 1×10^4^ E006AA or LNCaP cells were plated per well and allowed to adhere for 4 or 24 hrs, respectively. Cells were transduced at an MOI of 2 (2×10^4^ particles/well) without polybrene. Antibiotic selection [1.5 µg/ml puromycin (Sigma)] was initiated 48 hrs later.

### AR Sequencing

RNA was harvested using the Qiagen miRNeasy Mini kit (Qiagen, Valencia, CA) and contaminating DNA was removed using the Ambion DNA-free kit (Ambion, Austin, TX). Superscript reverse transcriptase III (Invitrogen, Carlsbad, CA) and 1 µg of total RNA were used to generate cDNAs with Oligo-dT primers. Genomic DNA was extracted using the Qiagen DNA Mini kit and incubated with RNase (Roche, Mannheim, Germany) at 37°C for 30 min to remove contaminating RNA. PCR amplification of cDNAs and genomic DNA was performed using Platinum *Pfx* DNA polymerase (Invitrogen) according to the manufacturer's specifications with the following overlapping primer sets: (1) 5′-AGAGAGGTAACTCCCTTTGGCT-3′ and 5′-ACGCTCTGGAACAGATTCTGG-3′ (740bp), (2) 5′-TCCCGCAAGTTTCCTTCTCT-3′ and 5′-GATACTGCTTCCTGCTGCTGTT-3′ (756bp), (3) 5′-TGAGGAACAGCAACCTTCACAG-3′ and 5′-ACAGGGTAGACGGCAGTTCAA-3′ (704bp), (4) 5′-GCCGAATGCAAAGGTTCTCT-3′ and 5′-TTGACACAAGTGGGACTGGGA-3′ (700bp), (5) 5′-CAGTTGTATGGACCGTGTGGT-3′ and 5′-CTACACCTGGCTCAATGGCT-3′ (723bp), (6) 5′-CGGAAGCTGAAGAAACTTGG-3′ and 5′-AGAAGCGTCTTGAGCAGGAT-3′ (685bp), (7) 5′-ATTCCAGTGGATGGGCTGAA-3′ and 5′-TAGCTCTCTAAACTTCCCGTGG-3′ (714bp), (8) 5′-CTTCCCATTGTGGCTCCTAT-3′ and 5′-ACCTTCTCGTCACTATTGGC-3′ (361bp); and for genomic DNA amplification of exon 3 of the AR gene: (9) 5′-TGTTTGGTGCCATACTCTGTCCAC-3′ and 5′-GCATCCTCACTCACCTTCTGTTGG-3′ (530bp). PCR products were column purified using the Qiagen QIAquick kit (Qiagen) and were sequenced at the Johns Hopkins University DNA Analysis Facility using both forward and reverse primers.

### Western Blot Analysis

Whole cell lysates were prepared on a per cell basis in lysis buffer (20 mM Tris-HCl pH 7.8, 140 nM NaCl, 1 mM EDTA, 0.5% sodium deoxycholate, 0.5% Nonidet P-40) supplemented with PhosSTOP phosphatase inhibitor tablet and protease inhibitor tablet (Roche), and 1 mM dithiothreitol. Whole cell lysates were subjected to SDS-PAGE and transferred to PVDF membranes. Membranes were blocked in 5% milk (TBS +0.1% Tween-20) for 1 hr. The rabbit polyclonal AR antibody (SC-816), and mouse monoclonal CK8 (SC-53266) and mouse monoclonal CK18 (SC-32722) antibodies were purchased from Santa Cruz Biotechnology (Santa Cruz, CA), and the mouse monoclonal ß-Actin antibody was purchased from Sigma (A5441) and were used in conjunction with an anti-rabbit-HRP antibody (7074, Cell Signaling Technology, Danvers, MA) or anti-mouse-HRP (NA931V, GE Healthcare, UK).

Nuclear and cytoplasmic cell fractions were prepared using the Nuclear Complex Co-IP kit (Active Motif, Carlsbad, CA) according to manufacturer's instructions. In brief, cells were washed and then scraped in cold PBS supplemented with phosphatase inhibitors. Cell pellets were resuspended and lysed in 1× hypotonic solution plus detergent, centrifuged at 6,000×g for 5 min at 4°C, and cytoplasmic fractions removed. Nuclear pellets were resuspended in digestion buffer plus enzymatic shearing cocktail and incubated at 4°C for 90 min. 0.5 M EDTA was added to stop enzymatic shearing reactions and nuclear lysates centrifuged for 10 min at 4°C. All fractions were combined with equal volume 2× SDS-sample buffer and denatured at 95°C for 5 min. Samples were separated on 4–15% PAGE and blotted with either the anti-AR (N-20; Santa Cruz), the cytoplasmic marker Vinculin (Sigma), or the nuclear marker HDAC (Santa Cruz Biotechnologies).

### Measuring AR Transcriptional Activity

Cells were plated (per well) in white polystyrene, clear bottom, sterile 96-well tissue culture plates accordingly: LNCaP C4-2B (9,000); PC-3 (7,000); E006AA, E006AA LV-AR, E006AA LV-non-silencing-shRNA, E006AA LV-AR-shRNA (5,000). Cells with or without androgen (1.0 nM R1881) were plated in six replicates in RPMI (Invitrogen) supplemented with 10% csFBS (Hyclone) but without phenol red or pen/strep and allowed to adhere overnight. Cells were transfected using Fugene 6 (Roche) with 50 ng Probasin-PSA-firefly and 5 ng pRL-TK-renilla (Promega, Madison, WI) Luciferase reporter constructs per well at a ratio of 3∶1 (µl Fugene: µg total DNA) in serum-free media [26]. On day three, six treatment wells were stimulated with androgen while six control wells received 0.1% vehicle. On day 4, media was carefully aspirated and the cells were processed according to the Dual Luciferase Assay kit instructions (Promega # E1910). Briefly, 30 µl of passive lysis buffer was added to each well and the plate agitated on a shaker for 15 min at room temperature. Firefly and renilla enzymatic activity was measured using a Wallac Jet 1450 Microbeta plate injector. Triple transfected LNCaP C4-2B cells received 50 ng Probasin-PSA-firefly, 5 ng pRL-TK-renilla, and 50 ng LV-AR-S599G constructs per well at a ratio of 3∶1 (µl Fugene: µg total DNA) in serum-free media.

### Chromatin Immunoprecipitation

LNCaP C4-2B and E006AA cells grown in phenol red-free RPMI, supplemented with 10% csFBS, were harvested via trypsinization and chromatin prepared according to the MagnaChIP kit from Upstate, Temecula, CA. ∼1×10^7^ cells were resuspended in 10 ml media, fixed by adding 275 µl of 37% formaldehyde and gently shaken at 22°C for 12 min. After crosslinking, 1 ml of glycine was added and cells gently shaken for 5 min at 22°C, washed twice in cold PBS, resuspended in 500 µl cell lysis buffer plus 2.5 µl protease inhibitors (5 mM PIPES pH 8, 85 mM KCl, 0.5% NP-40) and centrifuged for 5 min at 4°C. Cell pellets were resuspended in 500 µl cell lysis buffer plus 2.5 µl protease inhibitors, incubated 15 min on ice with brief vortex every 5 min, and centrifuged at 800×g for 5 min at 4°C. Nuclear pellets were resuspended in nuclear lysis buffer plus 2.5 µl protease inhibitors (1% SDS, 10 mM EDTA, 50 mM Tris-HCl pH 8.1). Chromatin was sonicated to an average DNA length of 500–1200 bp using Fisher Scientific Sonic Dismembrator Model 100 (4×15 sec pulses at setting 3). Five µl was saved for control input and 50 µl aliquots were diluted in 450 µl dilution buffer (0.01% SDS, 1.1% Triton, 1.2 mM EDTA, 16.7 mM Tris-HCl pH 8.1, 167 mM NaCl). Four µg of antibody was added to respective tubes: 4 µl of 1 mg/ml rabbit IgG or 40 µl 100 µg/ml rabbit anti-AR (SC-816, Santa Cruz) and rotated O/N at 4°C. Immune complexes were centrifuged 10 min at 10,000×g for 4°C to pellet any aggregates or non-specific complexes. The supernatant (desired immune complexes) was harvested and combined with 20 µl protein A Dynabeads (Invitrogen, CA) and gently stirred 2 hrs at 4°C. Tubes were placed in a Millipore magnetic rack and immune complexes sequentially washed 1 time with low salt buffer (0.1% SDS, 1% Triton, 2 mM EDTA, 20 mM Tris-HCl pH 8.1, 150 mM NaCl), high salt buffer (0.1% SDS, 1% Triton, 2 mM EDTA, 20 mM Tris-HCl pH 8.1, 500 mM NaCl), LiCl buffer, and TE, with 3 min stirring each wash step. To reverse the crosslinks, beads were resuspended in 100 µl of Chelex and boiled 10 min at 95°C, centrifuged 1 min at 12,000×g at 4°C, and supernatants collected. Beads were resuspended in 120 µl water, centrifuged again, and supernatants pooled with previous supernatant. The captured DNA was purified using the Qiagen PCR purification kit (#28104) according to manufacturer's instructions and analyzed by PCR and q-PCR.

### PCR Analysis of Immunoprecipitated DNA

Using 5 Prime HotMasterMix (Eppendorf), PCR reactions were performed as follows: 94°C for 2 min, then 32 cycles 30 sec denaturation at 94°C, 30 sec of annealing (as indicated below for each primer pair), and 30 sec of elongation at 65°C, then a 2 min final elongation at 65°C. PCR products were separated on 2% Agarose gels and stained with ethidium bromide. Primers for amplification of DNA fragments are as follows: PSA promoter ARE (52°C) fwd 5′-GCCAAGACATCTATTTCAGGAGC-3′, rev 5′-CCCACACCCAGAGCTGTGGAAGG-3′; KLK2 promoter (52°C) fwd 5′-CTCCAGACTGATCTAGTATG-3′, rev 5′-TTGGCACCTAGATGCTGACC-3′; SP1 site P45^Skp2^ promoter (53°C) fwd 5′-GATCCACGCTCAGAGACGAC-3′, rev 5′-TTCGAGATACCCACAACCCC-3′; Tcf4 binding element in c-Myc promoter (55°C) fwd 5′-GCTCTCCACTTGCCCCTTTTA-3′, rev 5′-GTTCCCAATTTCTCAGCC-3′.

### Quantitative PCR Analysis of Immunoprecipitated DNA

Using BioRad IQ SYBER Green Supermix (Hercules, CA), PCR reactions were performed with the BioRad ICycler as follows: 95°C for 3 min, then 40 cycles 30 sec denaturation at 95°C, 30 sec of annealing at 55°C, and 45 sec of elongation at 72°C (data were collected during extension), followed by a 110cycle melt curve from 45–100°C to ensure primer efficiency. Previously designed primers for quantitative amplification of DNA fragments containing a putative ARE in the PSA gene promoter and an ARE III in the PSA enhancer region are: ARE fwd 5′- CCTAGATGAAGTCTCCATGAGCTACA-3′, rev 5′-GGGAGGGAGAGCTAGCACTTG-3′; ARE III fwd 5′-TGGGACAACTTGCAAACCTG-3′, rev 5′-CCAGAGTAGGTCTGTTTTCAATCCA-3′ [27]. Percent input calculations were done as follows: % input  = 2(Ct_AR ChIP_ – Ct_input_)x100. Student's t-test was used to determine p-value.

## Results

### E006AA Cells are Tumorigenic in Intact and Castrated NOG-SCID Mice

**Figure 1:**
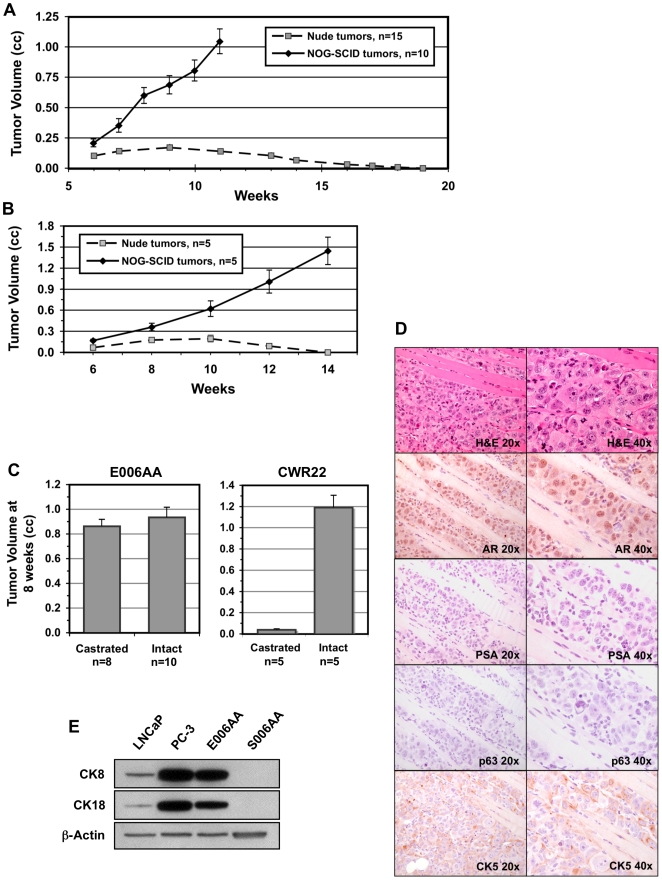
E006AA cells are tumorigenic and immunogenic in nude mice, and do not require a physiologic level of testosterone for growth. (A) Inoculation of E006AA cells into male nude mice compared to male NOG-SCID mice. (B) Transfer of tumor tissue derived from an established E006AA xenograft tumor from a male NOG-SCID mouse to intact male Nude and NOG-SCID mice. (C) The growth of E006AA and CWR22 human prostate cancer xenograft tumors in castrated vs. intact male mice at eight weeks. (D) Histological analysis (H&E) and immunostaining of E006AA xenograft tissue sections for AR, PSA, p63, and CK5 (images taken at 20× and 40×). (E) Western blot analysis of CK8 and CK18 expression in LNCaP, PC-3, E006AA and S006AA cells. ß-Actin served as a loading control.

Koochekpour et al. reported the establishment of a new human prostate cancer cell line, E006AA, which was derived from a Gleason 6 localized prostate cancer in a hormone-naïve prostate cancer patient of African American descent [20]. Matching normal prostate stromal cells, termed S006AA, were also cultured from the same radical prostatectomy specimen [20]. The epithelial origin of the E006AA cells was confirmed by positive expression for cytokeratins 8 (CK8) and 18 (CK18), whereas the stromal origin of the S006AA cells was confirmed by the positive expression of mesenchymal markers desmin and alpha-smooth muscle actin and the absence of CK8 and CK18 [20]. In the original studies, E006AA cells were non-tumorigenic when xenografted into intact male nude mice [20]. Since nude mice have abnormally high levels of activated natural killer (NK) T-cells, resulting in an increased host immuno-reactivity towards a growing tumor [28], this raised the question of whether these E006AA cells were immortalized but only partially transformed or whether they are fully malignant but sensitive to NK cell killing. To resolve this issue, E006AA cell subcutaneous inoculations into both intact male nude and NOG/SCID/γ_c_^null^ triple deficient mice [i.e., NOG-SCID mice deficient in NK, B, and T-cells] were initiated [29]. Inoculation of E006AA cells into nude mice (n = 15) demonstrated that E006AA cells form palpable tumors, reaching ∼100–200 mm^3^ by six weeks, in 100% of the mice. After six weeks, however, all of the E006AA tumors in intact male nude mice stopped growing and regressed over a second period of 5–10 weeks (Figure 1A). In contrast to the situation in nude mice, E006AA tumors grew at an accelerated rate in NOG-SCID mice and did not regress (Figure 1A). Interestingly, although tumorigenic, no serum PSA could be detected in E006AA tumor-bearing mice (n = 10).

To further confirm that the observed spontaneous regression of E006AA xenograft tumors in nude hosts, but not in NOG-SCID mice, stems from immune recognition and elimination by NK cells, we removed an established and growing E006AA tumor from a NOG-SCID host and transplanted tissue from this tumor back into either intact male NOG-SCID (n = 5) or nude mice (n = 5). Similar to our previous observations, transplanted E006AA tumors underwent delayed regression in intact male nude mice while forming continuously growing tumors in NOG-SCID hosts (Figure 1B). These combined results document that E006AA cells are indeed tumorigenic but vulnerable to NK cell killing.

Having demonstrated the tumorigenicity of E006AA cells, we next evaluated their androgen responsiveness, *in vivo*, by inoculating these cells into both intact (n = 10) and castrated (n = 8) male NOG-SCID hosts. Tumors developed equally well in both hosts and there was no difference in the growth rate or tumor volume in the castrated vs. intact NOG-SCID animals (Figure 1C). To ensure castrate levels of circulating androgens, the growth of the androgen-responsive CWR22 human prostate cancer xenograft in castrated (n = 5) vs. intact (n = 5) male mice was also analyzed. Unlike in intact mice, the growth of CWR22 xenograft tumors in castrated hosts was profoundly inhibited (Figure 1C). These results document that E006AA cells exhibit castrate-resistant growth, *in vivo,* even though they were originally established from a hormonally naïve primary prostate cancer. To characterize the phenotype of these E006AA cells, NOG-SCID xenograft tumors were removed and processed for histological and immunohistochemical analysis. Figure 1D illustrates that E006AA cells are locally invasive, penetrating skeletal muscle fibers at the local site of inoculation with the invading cells exhibiting hallmark features of enlarged and polymorphic cancer cell nuclei in which AR protein expression is detectable. In agreement with previous serum analysis, the E006AA xenograft cells did not stain positive for PSA (Figure 1D), or AR-regulated Nkx 3.1 proteins (data not shown). Additionally, the cancer cells lacked expression of the basal cell specific marker, p63, but were positive for epithelial cell markers cytokeratin 5 (CK5) (Figure 1D), CK8 and CK18 (Figure 1E), further confirming the epithelial origin of the E006AA cells [30], [31], [32].

**Figure 2:**
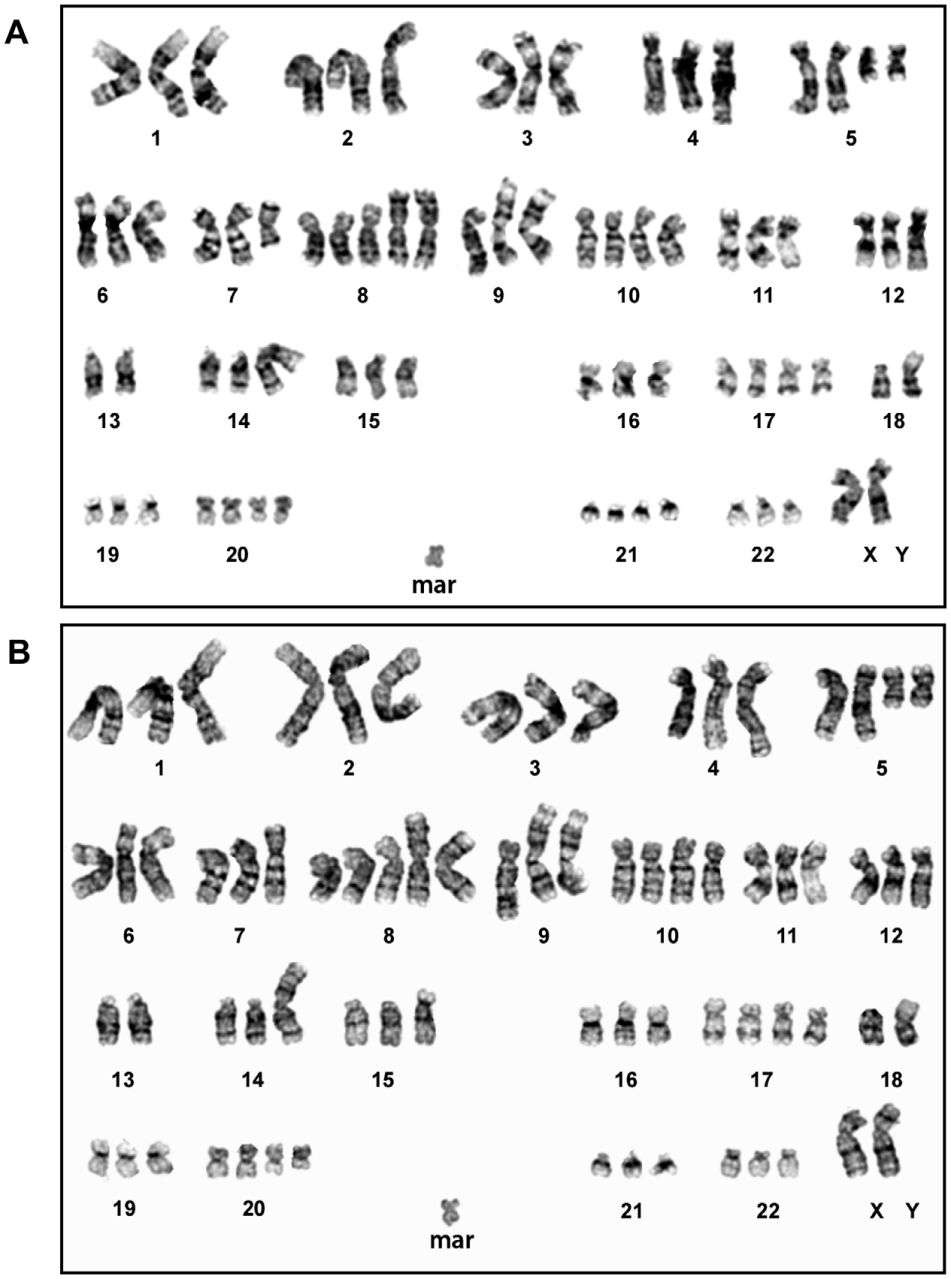
Karyotype Analysis of E006AA cells. (A) Karyotype analysis of E006AA cells prior to injection and *in vivo* tumor growth. (B) Analysis of E006AA cells derived from a NOG-SCID tumor-bearing mouse, documenting no significant karyotypic alterations during tumor growth.

To rule out an alternate explanation for why E006AA cells were shown to be tumorigenic in the present study, yet non-tumorigenic in the original studies, E006AA cells routinely maintained *in vitro* were karyotyped directly from cell culture (Figure 2A) and found to have an identical, abnormal karyotype as originally reported for the E006AA cell line [20]. Such cells were inoculated into NOG-SCID hosts and a continuously growing tumor was subsequently harvested to re-establish *in vitro* E006AA cultures. Karyotype analysis of these tumor-derived E006AA cells documented the same abnormal karyotype, as seen in the *in vitro*-maintained cell line (Figure 2B), eliminating the possibility that E006AA cells became genetically unstable after inoculation into NOG-SCID mice and developed additional cytogenetic changes that completed their malignant transformation into a tumorigenic variant.

### Amplification, Mutation, and Expression of AR in Castrate-Resistant E006AA Cells

**Figure 3:**
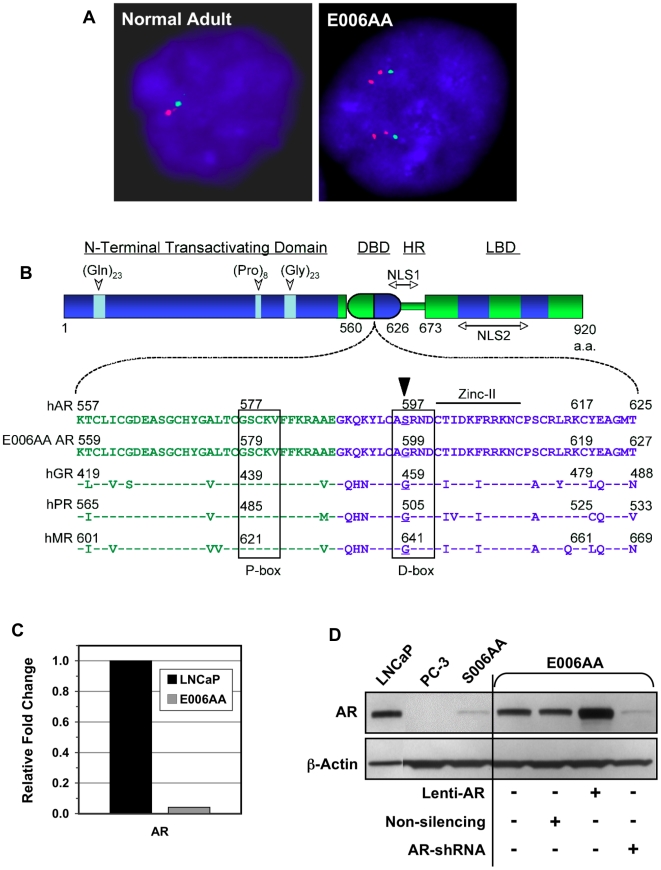
Somatic AR mutation and amplification in E006AA cells. (A) Cytogenetic analysis of AR status in normal male control and E006AA cells as determined by Fluorescence in Situ Hybridization (FISH) using probes specific for the AR Gene (Xq12, red colored) and the X centromere (green colored). (B) Sequencing results of overlapping primer PCR amplification of E006AA mRNA and cDNA identified a missense mutation (Ser599Gly), which corresponds to the “X” position of the conserved AXRND motif in the DBD D-box (as denoted by black arrowhead at position 597 of the reference human AR NM_000044), as found in human AR, GR, PR, and MR. (C) Quantitative PCR analysis of AR mRNA transcript levels in LNCaP compared to E006AA cells. (D) Western blot analysis of AR protein expression in E006AA cells infected with lentiviral non-silencing shRNA, wild-type AR (Lenti-AR), or anti-AR shRNA (AR shRNA) constructs compared to parental E006AA, LNCaP, PC-3 and S006AA stromal cells. Protein lysates were loaded on a per cell basis as follows: LNCaP (20,000), all others (200,000) cells per lane.

Fluorescence *in Situ* Hybridization (FISH) was performed with probes specific for the *AR* gene [Xq12] (red signal) and the X centromere (green signal) (Figure 3A). Interphase analysis demonstrated that in 91% of cells examined, there were two red signals associated with each green signal. Since there is a duplication of the X-chromosome, this means that, on average, the *AR* gene is amplified at least four-fold in the E006AA cell line.

Sequencing of overlapping primer PCR amplifications of E006AA AR cDNA revealed a single point mutation in the dimerization interface of the AR DNA binding domain (DBD) (Figure 3B). Sequencing also documented an overall 2,766 bp AR coding sequence consisting of a 27 aa polyglutamine tract (26 CAG repeats followed by one CAA), an 8 aa polyproline tract, and a 20 aa polyglycine tract. Koochekpour et al. originally reported 26 CAG repeats, which is in agreement with our analysis [20]. The point mutation is a missense mutation in the third exon consisting of a single nucleotide change from adenine to guanine at the 1795^th^ position (AGC to GGC). This single base change results in an amino acid substitution from serine to glycine at position 599 (S599G, Figure 3B). The S599G amino acid mutation corresponds with Ser597 in the reference AR protein sequence (NM_000044), due to an overall increase of two amino acids from the altered polyglutamine and polyglycine tracts. The significance of the S599G mutation pertains to its precise location at the AR dimerization interface and the documented role of this serine residue in supporting receptor dimerization when AR is bound to DNA [33], [34]. In other words, a serine-to-glycine change at this position, producing what is referred to as a “glycine hole”, disrupts the cross-dimer serine-serine hydrogen bond, which normally is crucial in promoting stability of the AR homodimer at the DNA interface.

To validate this single nucleotide change, each E006AA cDNA PCR product, generated from multiple cell passages, was sequenced using both sense and anti-sense primers. Additionally, genomic DNA was obtained from E006AA as well as from the patient-matched S006AA cells; PCR amplification using an intronic forward primer and an exonic reverse primer, designed to amplify only the mutation-containing region in exon 3, verified that E006AA cancer cells harbor the S599G mutation while the matching stromal S006AA cells do not. All sequencing reactions demonstrated an unambiguous signal at the mutation site, indicating that amplified copies of the *AR* gene within E006AA cells all harbor the same mutation. These data document that the *AR* gene mutation in E006AA was a somatic mutation that likely occurred prior to gene amplification and X-chromosome duplication.

Quantitative PCR analysis of AR mRNA in E006AA compared to LNCaP cells found that E006AA cells express approximately 23-fold lower levels of AR transcript than LNCaP cells (Figure 3C), but when compared to patient-matched S006AA normal prostate stromal cells, E006AA cells express a >10-fold higher level of AR protein (Figure 3D). However, it is important to note that when compared to other commonly used AR-expressing prostate cancer cell lines (i.e., LNCaP, LNCaP C4-2B, LAPC4), the levels of AR protein expressed by E006AA cells is approximately five- to ten-fold lower (Figure 3D) [13].

### Effect of Wild-Type AR Expression and shRNA AR Knockdown on E006AA Growth

**Figure 4:**
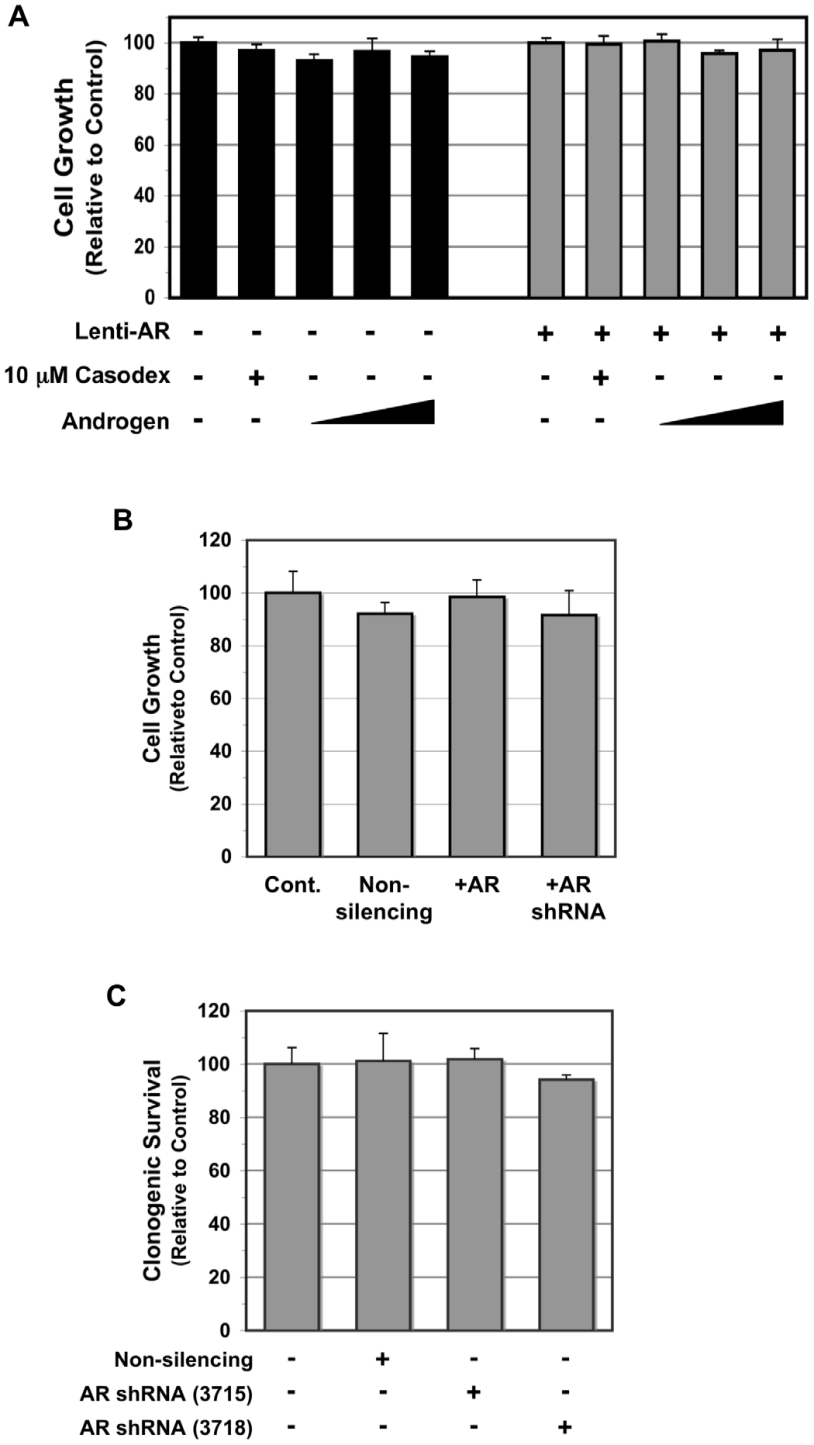
In vitro growth response to modulation of AR expression in E006AA cells. (A) Cell growth analyses of E006AA cells transduced with or without Lentiviral wild-type AR, which were then treated with 10 µM Casodex or an increasing dose of R1881 (1.0 nM to 100 nM). Cell growth is graphed relative to the respective untreated control cells. (B) Comparison of vector only Lentiviral-non-silencing, Lenti-AR, and Lenti-AR-shRNA transduced E006AA cell growth relative to control cells. (C) Colony forming assay comparing parental control cells to non-silencing, and two different anti-AR Lenti-shRNA sequences (3715 and 3718).

Paradoxically, while *AR* is mutated and amplified in E006AA cells, the *in vitro* growth of these cells, as determined by a cell growth and viability MTT assay normalized for cell counts, is not affected by the addition of an increasing dose of synthetic androgen (e.g., 1.0 to 100 nM R1881), or anti-androgen (e.g., 10 µM Casodex) (Figure 4A). Also, E006AA cells fail to produce detectable levels of secreted PSA protein (<0.1 ng/ml in media), which agrees with our *in vivo* serum and immunohistochemical analysis (Figure 1D) and, as reported originally by Koochekpour et al., E006AA cells do not express PSMA or PSAP [20]. One possibility is that this lack of ligand-dependent androgenic response could be explained by reduced stability of AR dimerization at the DNA interface due to the S599G mutation.

To begin resolving whether the lack of AR signaling is due to the mutant AR, wild-type AR (wt-AR) was introduced into E006AA cells via lentiviral transduction, to co-express ectopic wt-AR, and cells isolated via fluorescence activated cell sorting (FACS) for GFP-positive cells [8]. Such E006AA LV-AR sorted cells expressed ∼3∶1 ratio of wt-AR to mutant AR protein based upon comparison to parental cells expressing only mutant AR protein (Figure 3D). Even with such over expression of wt-AR protein, growth of E006AA LV-AR cells was not affected by the synthetic androgen R1881 or inhibited by the anti-androgen Casodex (Figure 4A). Interestingly, expression of wt-AR also failed to induce any detectable secreted PSA protein (<0.1 ng/ml in media) even in the presence of added R1881.

These results raise the issue of whether AR signaling is needed at all for the growth of E006AA cells. To address this, AR protein expression was suppressed using infection of anti-AR shRNA lentiviral particles. Western blotting confirmed a stable >95% knockdown of AR protein in these E006AA LV-AR-shRNA transduced cells (Figure 3D). Such AR protein knockdown, however, had no effect on either cell growth (Figure 4B) or clonogenic survival (Figure 4C). To verify the shRNA specificity for AR knockdown, transduction of LNCaP cells with the same AR shRNA lentiviral particles dramatically inhibited LNCaP cell growth and clonogenic survival (data not shown), as has been previously reported [35]. In conjunction with our *in vivo* castration studies, these data demonstrate that the growth of E006AA cells is not dependent on AR protein expression and function.

### Ligand-Bound AR Translocates to the Nucleus, Binds DNA, but is not Transcriptionally Active

**Figure 5:**
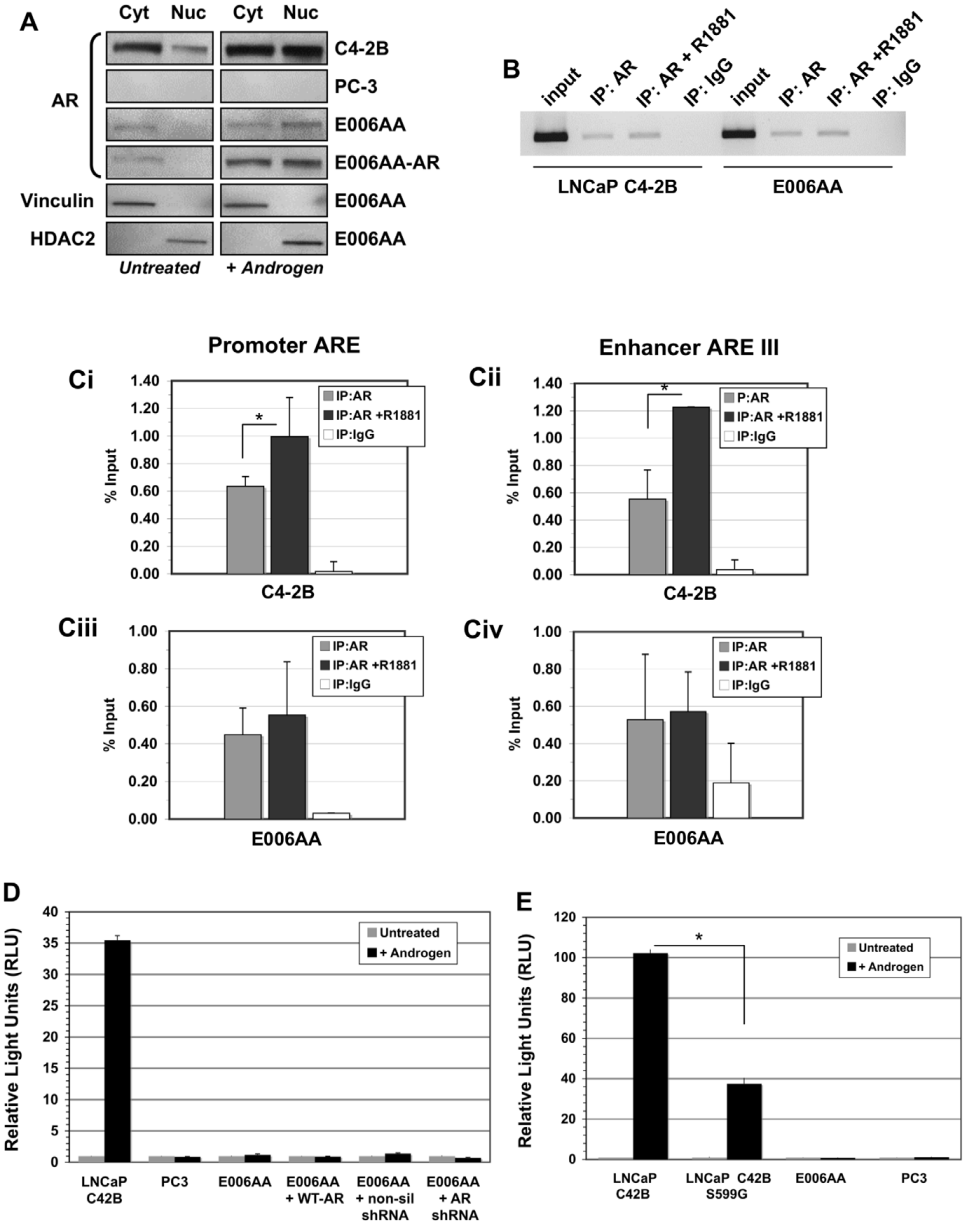
AR in E006AA cells undergoes nuclear translocation, binds DNA but is not transcriptionally activity due to the S599G loss-of-function mutation. (A) Western blot analysis of nuclear and cytoplasmic AR expression in LNCaP C4-2B, PC-3, and E006AA cells, treated with or without androgen. E006AA fractions were blotted for Vinculin and HDAC to confirm suitable cytoplasmic and nuclear separation, respectively. (B) PCR amplification of a putative ARE in the PSA promoter region from anti-AR immunoprecipitated chromatin from LNCaP C4-2B and E006AA cells, treated with or without androgen. Chromatin immunoprecipitated with an IgG isotype served as a negative control. (C) Quantitative PCR analysis of anti-AR antibody immunoprecipitated chromatin from LNCaP C4-2B and E006AA cells, treated with or without androgen: (Ci) PSA promoter ARE amplification in C4-2B cells; (Cii) PSA enhancer ARE III amplification in C4-2B cells; (Ciii) PSA promoter ARE amplification in E006AA cells; (Civ) PSA enhancer ARE III amplification in E006AA cells. The results are presented as percentage of input. (*, p-value <0.05, Student's T-test). (D) Analysis of AR transcriptional activity in E006AA cells compared to LNCaP C4-2B, PC-3, E006AA Lenti-AR, E006AA Lenti-non-silencing-shRNA, E006AA Lenti-AR-shRNA cells, treated with or without androgen. (E) Introduction of mutant S599G AR into androgen-responsive, PSA producing LNCaP C4-2B cells, and its effect on AR-driven PSA-Luciferase activity in C4-2B cells, with or without androgen (*, p-value <0.05, Student's T-test). All experiments in this section were performed with cells grown in 10% csFBS supplemented media, +/−24 hr stimulation with 1.0 nM R1881.

The inability of both wt-AR expression and targeted knockdown of the endogenous mutant AR to affect the growth of E006AA cells implies that the AR signaling machinery is deficient in E006AA cells. This could involve, among many possible mechanisms, impaired AR nuclear translocation or a loss-of-function due to the S599G mutation. To test this, the nuclear translocation and transcriptional activity of both the mutant and exogenously expressed wt-AR were analyzed. It has been well documented that, in the absence of ligand, AR is predominantly localized in the cytoplasm; however, upon binding ligand they localize to the nucleus where they bind DNA and activate target genes [1], [36], [37], [38]. E006AA cells, which were either depleted of or treated with androgen, were lysed and fractioned into nuclear and cytoplasmic fractions (Figure 5A). As controls, the castration-resistant and AR-positive prostate cancer cell line LNCaP C4-2B and the AR-negative prostate cancer cell line PC-3 were used. In agreement with previous studies [1], [37], [38], the majority of AR protein was detected in cytoplasmic fractions of both LNCaP C4-2B and E006AA cells grown in reduced androgen conditions (Figure 5A). Importantly, AR in LNCaP C4-2B cells as well as both the endogenous mutant AR and the lentiviral expressed wt-AR in E006AA cells translocates to the nucleus upon the addition of ligand (Figure 5A), documenting that E006AA cells possess the necessary cellular components to shuttle the AR into the nucleus upon ligand binding.

Knowing that endogenous, mutant AR in E006AA cells translocates to the nucleus upon stimulation with androgen, the next step was to evaluate whether the S599G mutant AR in E006AA cells had the capacity to bind DNA. Using an anti-AR antibody, chromatin was immunoprecipitated from E006AA and LNCaP C4-2B cells grown in reduced serum conditions (csFBS + phenol red-free RPMI) in the presence or absence of androgen. The captured DNA was subjected to semi-quantitative PCR amplification using primers designed to amplify putative androgen response elements (ARE) located in the PSA promoter. Positive amplification is detected in AR-immunoprecipitated chromatin from both E006AA and C4-2B cells, demonstrating that the endogenous, mutant AR expressed in E006AA cells binds DNA, regardless of the presence of ligand (Figure 5B). To test a wider array of AR-binding sites, PCR analysis was performed using primers targeting the AR-regulated Sp1(A) site in the human p45^Skp2^ promoter [39], the AR binding site in the human KLK2 promoter [40], and the AR-associated Tcf4 binding element in the c-Myc promoter [41], all of which demonstrated unambiguous amplification of AR-immunoprecipitated chromatin (data not shown). Additionally, quantitative PCR, using primers designed to amplify a similar ARE-containing region in the PSA promoter [27] and a putative androgen response element III (ARE III) located in the PSA enhancer [27], was performed to evaluate the effect of androgenic stimulation on the DNA binding ability of mutant, endogenous AR in E006AA cells. As seen using semi-quantitative PCR, AR from both LNCaP C4-2B and E006AA cells was detected at the PSA promoter (Figure 5Ci, Ciii). Surprisingly, we observed positive amplification at the PSA enhancer region from both LNCaP C4-2B and E006AA immunoprecipitated chromatin samples, regardless of ligand stimulation (Figure 5Cii, Civ). However, this experiment revealed that, although mutant AR in E006AA cells can be found at the promoter ARE and enhancer ARE III sites, androgenic stimulation failed to increase S599G AR presence at either site, in contrast to what was observed in the LNCaP C4-2B cells (Figure 5C).

To test whether endogenous AR in E006AA cells possesses the capacity for AR-mediated gene transcription, transient transfection assays were performed using a Probasin-PSA-Firefly Luciferase vector, which is optimized for measuring AR transcriptional activity [26]. Using steroid-depleted, serum-containing media, Luciferase activity was tested in the absence and presence of exogenous androgen. Endogenous, mutant AR in E006AA cells failed to drive Luciferase expression from the AR-mediated promoter construct (Figure 5D). In agreement with the previous finding that E006AA LV-AR cells did not secrete detectable PSA, introduction of wt-AR also failed to induce Luciferase expression (Figure 5D); previous data have documented that such wt-AR expression is capable of driving AR-mediated gene transcription in appropriate cells [8].

The earlier demonstration that S599G mutant AR in E006AA cells translocates to the nucleus upon androgenic stimulation, where it effectively binds ARE-containing DNA but does not stimulate transcription of AR target genes, raises the issue of whether the S599G mutation results in a loss-of-function. To evaluate this possibility, the S599G mutant AR was cloned and placed in a Lentiviral vector to create a LV-AR-S599G construct. We then assessed Luciferase activity in the positive control LNCaP C4-2B cells transiently co-transfected with the LV-AR-S599G and Probasin-PSA-Firefly Luciferase vectors. In LNCaP C4-2B cells transiently expressing S599G AR, Luciferase activity was reduced 63%, in multiple experiments, compared to parental LNCaP C4-2B cells (Figure 5E). These combined results document that the S599G mutation is a dominant-negative loss-of-function mutation that disrupts normal AR signaling.

## Discussion

Prostate cancer cells can be subdivided based upon AR protein status and response to castration. In one subtype, AR protein is not expressed (e.g., PC-3 and DU-145) and thus cell survival and proliferation is completely independent of AR signaling. These AR negative cells are thus castration resistant. In another subtype of prostate cancer cells (e.g., PC-82 and CWR22), AR is expressed and has acquired a gain-of-function such that it no longer inhibits but now stimulates proliferation when activated by physiological levels of androgen [13]. Thus, this subtype of AR-expressing prostate cancer cells remains castrate-sensitive. In a second subtype of AR-expressing prostate cancer cells derived from castrated patients (e.g., LNCaP, LAPC-4, MDA-PC-2B, VCAP, etc), the cells have developed castration resistance mechanisms (i.e., mutation, gene amplification, protein over-expression) to activate AR signaling for their continued growth even in the presence of castrate levels of androgen [7].

E006AA cells represent a third new subtype of AR-expressing prostate cancer cells, which exhibit castrate-resistant growth characteristics even though these cells were derived from a hormonally naïve patient. E006AA cells possess many of the same molecular characteristic changes commonly observed in prostate cancer metastases and cell lines derived from patients who were androgen ablated. These include AR gene amplification and mutation. The significance of the S599G mutation is its precise location within the DBD, and more specifically in the dimerization interface region known as the D box [34]. Originally characterized from the co-crystal structure of the glucocorticoid receptor (GR) DBD bound to an inverted-repeat 4 DNA element (GR-DBD-IR4), the D-box loop structure contains a conserved AXRND motif within the second zinc-finger of the DBD [42]. The D-box sequence found in the GR, progesterone receptor (PR), and mineralocorticoid receptor (MR) contains an AGRND motif whereas AR contains an ASRND motif (Figure 3B). A change from serine to glycine at the “X” position of the mutated AR in E006AA cells destroys the crucial serine-serine hydrogen bond, which is thought to increase the stability of the AR homodimer at the DNA interface, resulting in what is referred to as a “glycine hole” (Figure 3B) [33]. If such a substitution sufficiently impairs the AR:DNA interaction [33], this loss-of-function mutation might explain the apparent lack of AR responsiveness observed in E006AA cells. The results presented here, which demonstrate that endogenous mutant AR in E006AA cells interacts with ARE-containing DNA, that E006AA AR fails to drive an AR-regulated luciferase construct, and that the S599G mutant AR, when cloned into an AR-responsive cell line that we know possesses all the necessary PSA transcription co-factors, profoundly inhibits endogenous AR transcriptional function, implicate the S599G mutation as a dominant-negative mechanism sufficient in shutting down the AR signaling axis in E006AA cells. Interestingly, this specific S599G substitution has been identified in male patients with androgen insensitivity syndrome (AIS) [43], [44] and is catalogued in the McGill University's *AR* gene mutations database for AIS [http://androgendb.mcgill.ca], but has not been previously linked with prostate cancer. This mutation, however, does not disrupt normal androgen binding kinetics [44], which agrees with our data showing that androgens can drive endogenous, mutant AR into the nucleus.

The defining characteristic of E006AA cells is the complete absence of AR growth-regulatory function, even though these cells express AR protein. This raises the issue of why such molecular changes in AR (i.e., gene amplification and a loss-of-function mutation) would have a selective advantage. One explanation is that while these changes prevent AR signaling from promoting either survival or proliferation of malignant prostate cells, the lack of AR signaling eliminates the growth suppression normally induced by AR signaling in non-transformed prostate epithelial cells in a host with normal physiological levels of androgen. This is consistent with the loss of AR-mediated growth suppression providing a selective growth advantage, even without the simultaneous acquisition of a gain-of-function ability of AR signaling to stimulate survival and proliferation. This suggests that the biochemical mechanism for loss of AR signaling-induced growth suppression is not identical, and not always coupled, to that associated with the gain of AR signaling-induced growth stimulation in prostate cancer cells. The inability to express AR regulated genes, such as PSA, Nkx3.1, and KLK2, could also be due to alterations in the AR transcriptional machinery in E006AA cells, in addition to possible epigenetic changes that have altered the chromatin configuration found in E006AA cells. These possibilities raise important questions for future studies and defining these distinguishable mechanisms is a focus of our ongoing experiments, for which E006AA cells are an important reagent.

Regardless of the mechanism, the realization that loss of growth suppression and gain of survival and proliferation stimulation by AR signaling are distinct processes has significant clinical implications. For example, as many as 50% of patients with castrate-resistant metastatic prostate cancer have circulating cancer cells that have substantial amplification of the AR locus, which has been interpreted to predict that, in such patients, androgen signaling continues to play an important growth stimulatory role [45]. The present data show, however, that this is not always true, since expression of mutated and amplified AR within E006AA cells serves no stimulatory function for either survival or proliferation. These results have a series of clinical implications: 1) AR gene mutation, amplification, or altered expression do not occur only following androgen ablation, 2) such molecular changes do not always result in AR signaling being required for either the survival or proliferation of prostate cancer cells, and 3) detection of such AR changes cannot be used absolutely to predict clinical response to AR axis-targeted therapy. An important clinical corollary to these conclusions is that there exists a subtype of prostate cancers for which a more aggressive hormone therapy regimen or anti-AR therapy will elicit no clinical benefit, even though they possess AR mutation or amplification.
